# The Need for Health Education and Vaccination—Importance of Teacher Training and Family Involvement

**DOI:** 10.3390/healthcare10010110

**Published:** 2022-01-06

**Authors:** Eduardo García-Toledano, Emilio López-Parra, Antonio Cebrián-Martínez, Ascensión Palomares-Ruiz

**Affiliations:** Department of Pedagogy, Universidad de Castilla-La Mancha, 02071 Albacete, Spain; toledanoeg@gmail.com (E.G.-T.); emilio.lopezparra@uclm.es (E.L.-P.); antonio.cebrian@uclm.es (A.C.-M.)

**Keywords:** COVID-19, equity, family, health education, prevention, teacher training, vaccination

## Abstract

The health emergency due to COVID-19 has highlighted the importance of vaccination and its impact on social welfare. Inequalities have surfaced that affect the most vulnerable and those millions of children do not receive the necessary vaccines. Health education becomes a fundamental resource for citizens to access universal rights. One thousand people from 76 countries on five continents participated in this research in 2019–2020, from the health, education, and economic sectors. A descriptive cross-sectional study with a quantitative design was used. The instrument used was a correctly validated questionnaire: VACUNASEDUCA. The objectives were to reflect on the adequacy of teacher training and their awareness for the proper use of vaccines and to analyze the knowledge of parents about the consequences of vaccination. The results demonstrate the importance of teacher training and health education, with positive involvement of the family. The most favorable group is female, under 30 years, from the European continent, with a very high Human Development Index (HDI), and from the education sector. In conclusion, it is noted that, within the framework of the fourth industrial revolution, education must be configured with innovative approaches and tools, making it necessary to intervene in the context considering their cultural characteristics and promoting healthy lifestyle habits.

## 1. Introduction

The health emergency caused by the COVID-19 pandemic, declared by the World Health Organization (WHO) in March 2020 [1], and its subsequent spread across five continents, has led to important social, economic, and educational changes, demonstrating the importance of vaccination. Since then, it has generated a novel collaboration between countries and a marathon competition between pharmaceutical companies to achieve an effective vaccine. Vaccines have been recognized as one of the most effective tools to prevent the spread of disease [2]. Nowadays, vaccines are antigenic preparations with the ability to trigger an immune system response generating a long-lasting protection against a disease [3,4].

Since the creation of the Expanded Program on Immunization by WHO in 1974, there has been an explicit recognition of the importance of vaccination and its great impact on social welfare. As a result, a Global Vaccine Action Plan (GVAP) has been adopted (2011–2020) [5], achieving a common framework for setting priorities, agreeing on activities, and evaluating outcomes. Consequently, taking into account the lessons learned from the GVAP and the challenges posed by infectious diseases, the Immunization Agenda (IIA2030) has developed a global strategy on vaccines and immunization for the decade 2021–2030. It aims to strengthen existing partnerships and establish new relationships, better clarify roles and responsibilities, and improve the use of information to optimize assessments [1].

The global pandemic of COVID-19 prompted authorities to recommend confinement as a strategy to prevent and safeguard the health of all people. This resulted in the paralysis of “non-essential” activities such as socialization, employment, productivity, public health and, especially, the education sector, the consequences of which continue to affect the lives of citizens. All over the world, those responsible for education took measures to be able to continue school activities by drawing up corresponding emergency plans. In the past, various natural or social phenomena have led to the closure and interruption of national and local education systems. However, school activities have never been suspended for more than 1.5 billion students at different educational levels and around the world [6,7,8,9,10]. Teachers, students, and families have had to transform the dynamics of teaching–learning processes and work under parameters for which they were not prepared [11,12,13].

The problems have been further accentuated by the lack of equity in access to educational and social services. Therefore, international action organizations such as the United Nations Educational, Scientific, and Cultural Organization (UNESCO), WHO, the United Nations International Children’s Emergency Fund (UNICEF), the Council of Europe, the Organization for Economic Cooperation and Development (OECD) and the European Commission, insist on the need for schools to include health education as a key tool for developing healthy life habits and increasing the quality of life of schoolchildren [14].

Childhood vaccines save about three million lives a year by providing them with the antibodies needed to fight against very dangerous diseases such as measles, polio, or pneumonia [15]. However, today, millions of children do not receive vaccines, either because their parents refuse or because they do not have access to them. Similarly, if a child is not properly vaccinated, not only is his or her life at risk, but it also affects other children living with him or her, their families, and teachers. In fact, vaccines are synonymous with education because they improve their quality of life and their schooling process. However, regardless of socioeconomic or educational level, there are many people who question the existence of the virus; others defy social distancing measures, valuing them as very severe and ineffective; and there are even those who, despite knowing the danger, have to assume the risk and work (essential activity personnel). The refusal of vaccination, with different nuances, has been called “vaccine reluctance” [16,17].

The rejection of vaccination is occupying wide spaces of debate in the media and social networks, in multiple areas of society and in all countries of the world. At the same time, the consolidation of vaccine reluctance is being valued as a threat to collective health [18,19,20]. In this regard, the recommendations proposed by the WHO [16] focus on the need to better understand vaccine reluctance, its determinants, and the challenges it poses. Furthermore, it stresses the need to improve society’s awareness of the importance of vaccination to improve vaccine acceptance, share effective practices, develop new tools to assess and address reluctance. Reasonably, it is essential that teachers and families are aware of the benefits and efficacy of vaccines, concerns about their safety, and how they are perceived in society [21,22]. In fact, as Matesanz [23] points out, having an effective vaccine is not an individual matter, but what is important is that the maximum number of people in the environment receive it in order to achieve the desired “herd immunity” and stop the virus from circulating.

Health education is one of the main tools for societies and citizens to access universal rights. In 1983, the WHO considered ‘health education’ as a discipline focused on guiding and organizing educational processes, through a combination of information and education activities. The aim is to generate a scenario in which people yearn to be healthy, know how to achieve health, act individually and collectively to maintain it and, when needed, seek the help they require.

Health is considered to be the ability to develop one’s personal potentialities and respond positively to the challenges of the environment [24]. Reasonably, health education has a multidimensional perspective that facilitates knowledge, attitudes, and skills, instilling awareness of the determinants of health. In all processes, targeted learning should be facilitated to bring about changes in health-damaging behaviors or to maintain healthy ones.

Health education is an important tool through which to motivate change, discern the validity of the information received, establish communication channels, and empower individuals and communities to become activists in individual, environmental, and organizational redesign with globalizing actions. Therefore, its ultimate goal is the transformation of harmful behaviors and the reinforcement of healthy ones, and its fundamental axis is communication, encompassing aspects related to education, training, research, legislation, policy coordination, and communicative development [25,26]. In the literature, low educational levels have been associated with greater health problems [27,28], since a low level of health education can have its origin in various social barriers that hinder access to health services, difficulties in the correct use of medications, problems of access to adequate health information, or complications in the control of chronic diseases [29].

In this complex context, it is necessary to highlight the importance of promoting public health policies that implement health education programs with special attention to vaccination processes among the most vulnerable groups and the population in general. Consequently, it is essential that every member of any social class, ethnicity, and locality understand that infectious diseases are an important cause of morbidity and mortality, and that vaccination is the best tool for their prevention and reduction.

The research reflected in this article began in June 2019 and ended in September 2020, coinciding with the declaration of health emergency caused by the virus (SARS-CoV) causing the disease COVID-19. The work was initiated in view of the need to promote actions that favor the vaccination processes of all citizens of the world as a tool for the preservation of individual and collective health, and the strengthening of the global health system. The objectives of the research are to reflect on the adequacy of teacher training and their awareness of the proper use of vaccines, and to analyze the perception of society on the information that parents have about the consequences that may result from their children living with non-vaccinated peers.

## 2. Materials and Methods

A descriptive study with a quantitative cross-sectional design was carried out to study the importance of vaccination in the health of the child population. This article will analyze dimension D2: Education and Teachers, composed of the following four items:

- P03. Do you consider that, in your country, teacher training in compulsory pre-school and primary education provides adequate training on vaccines?
- P04. Do you appreciate that teachers are aware of the proper use of vaccines?
- P05. Do you think parents know the consequences that coexistence with other non-vaccinated peers could have for their children?
- P06. Do you believe that teachers at mandatory levels should receive initial training in health education and specifically in the vaccination process?

### 2.1. Population and Sample

The population under study is determined by the inclusion criterion of the exercise of their profession in the education, health, and economic sectors. By virtue of this criterion, the sample was selected by means of a non-probabilistic method of consecutive type or total enumerative sampling. A final sample of 1000 participants belonging to 76 countries from the five continents, whose characteristics according to Sex, Age, Sector, Human Development Index (HDI), and Continent are detailed in Table 1, was established.

It should be noted that the HDI was derived from the list of countries by human development index included in the United Nations Development Programmer’s (UNDP) Human Development Report 2020, published on 15 December 2020, and compiled based on 2019 estimates. It includes 189 United Nations member states (out of a total of 193) plus Hong Kong (special administrative region of China) and the State of Palestine. Missing member countries are due to lack of data required for the calculation. For comparison, the average HDI of world regions and country groups is also included.

### 2.2. Instrument

Data collection was carried out by means of the survey technique, using the VACUNASEDUCA questionnaire [30], which was developed ad hoc and designed to know the perception about of the importance, awareness, and mandatory nature of vaccines in the health of the child population in certain social sectors. The questionnaire consists of 12 items with a Likert scale. These items are distributed in four dimensions, two items corresponding to dimension D1 = Awareness and Regulation, four items corresponding to dimension D2 = Education and Teachers, two items corresponding to dimension D3 = Regulation and Obligation, and four items corresponding to dimension D4 = Consequences and Risks.

This questionnaire was subjected to a validation process by expert judgment through which the Content Validity Index (CVI) was calculated. The results obtained were for dimension D1 = 0.87; for dimension D2 =0.93; for dimensions D3 and D4 = 1 respectively. The mean index was 0.96.

On the other hand, the Kaiser–Meyer–Olkin (KMO) test was performed, in which a result of 0.784 was obtained, and Bartlett’s test of sphericity, in which a significance level of <0.001 was obtained, corroborating the adequacy for factor analysis.

In relation to the reliability of the instrument, Cronbach’s alpha (∝) was calculated, obtaining a mean result for the four dimensions of 0.64, close to the 0.70 established for acceptable consistency [31].

### 2.3. Variables

Each item is constituted in an ordinal variable, calculating the dependent variable of quantitative type S3t through the sum of each individual score of each participant and dividing by 12 to proceed with its typification. The variables of the study are:

- Sex: G0 = Woman or G1 = Man.
- Age: E1 ≤ 30, E2 = Between 30 and 44, E3 = Between 45 and 59, E4 ≥ 60.
- Sector: S1 = Health, S2 = Education, S3 = Economy.
- Human Development Index (HDI): I1 = Very High, I2 = High, I3 = Medium, I4 = Low.
- Continent: C1 = Europe, C2 = America, C3 = Asia, C4 = Africa, C5 = Oceania.

### 2.4. Procedure

The data collection was carried out between the months of September and December 2019 in the WHO office in Geneva (Switzerland), and from January to March 2020 in different centers and institutions located in Spain, such as hospitals, universities, congresses and meetings of education and medicine, among others.

The questionnaire was always applied by the same researcher in person, and was completed in a self-administered manner, in Spanish and English. No time limit was established for completion, although participants usually took between 5 and 10 min to complete it. Before completing the questionnaires, respondents were provided with sufficient and understandable information on the research topic, guaranteeing the anonymity and confidentiality of each respondent’s data.

#### Data Analysis

The sample elements did not meet the conditions established to be considered a normal distribution. Therefore, statistical techniques of null models were used through resampling techniques using the Monte Carlo method, using the Bootstrap procedure [32] thanks to current computer solutions and the large sample size to provide relevant information of the population to which it belongs [33].

An ANOVA test was performed for independent samples for each of the independent variables or research factors to check for statistically significant differences. Through the analysis of the Multivariate General Linear Model, the value of the F-statistic, the level of significance *p* and the size of the effect measured by eta squared were obtained. Non-equal variances were assumed using Tamhane’s T2, Dunnett’s T3, Games–Howell and Dunnett’s C in the post hoc tests, with similar results that determined the direction column in the ANOVA tables of each factor.

In addition, a nonparametric bivariate correlational analysis was performed among the study variables using the Spearman and Kendall tau tests, which yielded very similar results.

## 3. Results

The results are specified according to the objectives set for this research.

In this study we will focus on the data obtained in the dimension D2 = Education and Teachers with the variables Sex, Age, Sector, HDI, and Continent. The results obtained in this dimension are from a score of M = 2.81 and SD = 0.31, highlighting item P06 by obtaining a mean score higher than the rest (M = 2.86 and SD = 0.44). The descriptive statistics obtained are detailed in Table 2.

### 3.1. Sex Impact Analysis

The Sex distribution of the sample is uneven, with 69.4% of respondents being women and the remaining 30.6% being men (see Table 3).

In the ANOVA carried out to analyze the differences in relation to Sex, the results shown in Table 4 were obtained.

Post hoc tests indicate that, in dimension D2 = Education and Teachers, the mean of women is higher than that of men, with higher means and indicating a trend towards YES. Consequently, it can be inferred that, in general, women express themselves with higher means than men when they assess that teachers have adequate training and are aware of the use of vaccines and believe that teachers should receive initial training in health education and vaccination. Likewise, women obtain higher means than men when assessing whether parents are aware of the consequences that coexistence with other non-vaccinated peers could have for their children.

There are statistically significant differences in items P03, P04, P05, and P06, as well as in dimension D2, although the effect size measured by ANOVA per eta squared must be considered weak as it is less than 0.06 [34].

### 3.2. Analysis of the Incidence of Age

The sample distribution according to the age groups shows some inequality, since, while the groups E1 ≤ 30 years (36.3%), E2 = Between 30 and 44 years (34.8%), and E3 = Between 45 and 59 years (26.76%) present similar percentages, that of the group E4 ≥ 60 years (2.2%) is considerably lower (see Table 5).

In the ANOVA performed to analyze the differences in relation to the age group, the results reflected in Table 6 were obtained.

The post hoc tests carried out indicate that, in the dimension D2 = Education and Teachers, the mean of the age group E1 ≤ 30 years is higher than the other three groups, with higher means and indicating a trend towards YES. Therefore, it can be inferred that, in general, the age group E1 ≤ 30, is the one that most values the existence of teacher training and their awareness of the use of vaccines, as well as the need for them to receive initial training on health education and vaccination at mandatory levels. In addition, it is the age group E1 ≤ 30, which obtains higher means when assessing that parents know the consequences that coexistence with other non-vaccinated peers could have on their children.

There are statistically significant differences in items P03, P04, P05, and P06, as well as in dimension D2, although the effect size measured by ANOVA per eta squared must be considered weak as it is less than 0.06 [34].

### 3.3. Analysis of the Impact of the Sector

The distribution of the sample according to professional sector is unequal, with the highest percentage of participants belonging to the health sector (55.4%), 32.9% belonging to the education sector, and 11.7% to the economic sector (see Table 7).

In the ANOVA carried out to analyze the existing differences in relation to the professional sector, the results shown in Table 8 were obtained.

As can be seen, the post hoc tests performed indicate that, in dimension D2 = Education and Teachers, the mean of the education sector is higher than that of the health sector, with higher means and pointing to a trend towards the YES.

The means of the economy sector are in an intermediate position, since they show significant differences with the education sector and non-significant differences with the health sector in items P04, P05, P06, and in dimension D2. On the other hand, in item P03 the trend is reversed, so that the mean differences of the economic sector are not statistically significant with respect to the education sector, but they are statistically significant with respect to the health sector.

Reasonably, it can be inferred that, in general, respondents in the education sector value more highly the existence of teacher training and their awareness of the use of vaccines, as well as the need for them to receive initial training on health education and vaccination at the mandatory levels. Likewise, it is the education sector group, that obtains highest means when assessing that parents know the consequences that coexistence with non-vaccinated peers could have on their children.

There are statistically significant differences in items P03, P04, P05, and P06, as well as in dimension D2, although the effect size measured by ANOVA per eta squared must be considered weak as it is less than 0.06, while in dimension D2 it can be considered with a medium effect as eta squared is higher than 0.06 [34].

### 3.4. Analysis of the Incidence by Human Development Index (HDI)

The sample distribution according to the HDI is unequal, since the highest percentage of participants belongs to group I1 = Very high (87.3%), being represented the group I2 = High by 8.5%, the group I3 = Medium by 3.1% and the I4 = Low by 1.1% (see Table 9).

In the ANOVA performed to analyze the differences in relation to the HDI, the results shown in Table 10 were obtained.

The post hoc tests performed show that, in dimension D2 = Education and Teachers, the mean of the HDI group I1 = Very High, is higher than the rest of the groups, with higher means and indicating a trend towards the YES. Thus, it can be inferred that, in general, participants belonging to the HDI group I1 = Very High obtain higher means than the other HDI groups when assessing the adequate teacher training and awareness of the use of vaccines, as well as the need for them to receive initial training on health education and vaccination at mandatory levels. Likewise, it is the HDI I1 group, that obtains the highest scores when estimating that parents know the consequences that coexistence with other non-vaccinated peers could have on their children.

There are statistically significant differences in items P03, P04, P05, and P06, as well as in D2 dimension D2, although the effect size measured by ANOVA per eta squared must be considered weak, since it is less than 0.06 in items P05 and P06, while in items P03 and P04, eta squared is greater than 0.06, which is considered a medium effect [34]. In dimension D2, eta squared has a value greater than 0.14, so it can be considered a large effect [34].

### 3.5. Analysis of the Incidence by Continent

The sample distribution according to the Continent is unequal, since the highest percentage of participants belongs to C1 = Europe (83%), while the percentage of the rest of the continents is C2 = America (9.3%), C3 = Asia (4%), C4 = Africa (3.5%) and C5 = Oceania (2%) (see Table 11).

In the ANOVA carried out to analyze the existing differences in relation to the Continent, C5 = Oceania was excluded from the analysis, since the small number of participants from that continent prevents an adequate statistical analysis through the resampling techniques used in this study. The results obtained are shown in Table 12.

The post hoc tests carried out indicate that, in dimension D2 = Education and Teachers, the mean of the participants of C1 = Europe is higher than the rest of the continents, with higher means and indicating a trend towards the YES. Consequently, it can be inferred that, in general, respondents from the C1 = Europe continent offer higher means than the rest of the groups from other continents when assessing adequate teacher training and awareness of the use of vaccines, as well as the need for them to receive initial training on health education and vaccination at mandatory levels. Likewise, it is the group that has the highest scores when estimating that parents know the consequences that coexistence with other non-vaccinated peers could have on their children.

There are statistically significant differences in items P03, P04, P05, and P06, as well as in dimension D2, although the effect size measured by ANOVA per eta squared must be considered weak as it is lower than 0.06 in items P05 and P06, while in items P03 and P04 and in dimension D2 it can be considered a medium effect as eta squared is higher than 0.06 [34].

### 3.6. Correlational Analysis

Table 13 includes Spearman’s matrix of nonparametric bivariate correlations, which shows a significant positive correlation between the variables SEX, AGE, HDI, CONTINENT, and a significant negative correlation of the variable SECTOR with the other variables of the study. All correlations, when presenting a correlation coefficient between 0.10 and 0.30 can be considered with a small effect size, except the correlation between the HDI variables, CONTINENT that when presenting a correlation coefficient greater than 0.50 can be considered a large effect size [34].

## 4. Discussion

The acceleration of technological changes affecting society, the economy and employment, and the consequent flowering of a complex set of opportunities and risks for citizens, organizations, and governments, make the decisive role of education and training in reducing the threats, and in implementing possibilities for economic development and employment more visible.

The fact that women show less reluctance to vaccination than men [35] may explain the higher score obtained by women when evaluating teacher training and a greater perception of parental awareness of the importance of vaccines. However, there are discrepancies regarding the influence of the sex variable on the perception of the usefulness and importance of vaccines [35], and this relationship has not been found in other studies [36]. The consequences caused by the global pandemic have highlighted the need for greater investment in education and biomedical areas. The effects of the SARS-CoV pandemic have been found to be closely related to the level of investment and development (R&D), so that one of the factors causing the epidemic to advance is the low number of vaccinated populations worldwide and their uneven distribution in these countries. Keep in mind that the ability to achieve group immunity only works for vaccine-preventable diseases, showing that vaccines are a profitable investment and promote an improvement in the quality of life, especially in the most disadvantaged countries. Therefore, as González-García [37] states, it is essential to invest more in research to generate strength in the face of possible biological threats and pandemics that may occur.

At the beginning of the pandemic, students at all levels of education in developed countries have not been greatly affected in the area of health. However, because education systems around the world are not prepared to respond adequately, containment measures adopted by governments have influenced their physical and mental health, nutrition, leisure, response to their schooling, attention to diversity, etc. [10,11,38].

The fact that participants belonging to the HDI group I1 = Very High obtain higher scores when assessing teacher training and parental awareness of vaccines, is in line with what has been repeatedly pointed out by Swaminathan [39], chief scientist of the WHO: that in countries with lower Development Index (HDI), the consequences are very serious, giving rise to situations such as mistreatment, violence, abuse, exploitation, and, especially, interruption of vaccination processes, etc. [40,41,42]. Consequently, the crisis has aggravated the existing problems associated with the precarious conditions in which many families and children live. Logically, the negative effects have been greater on children in countries with fewer socioeconomic resources and, at the same time, on those affected with personal problems such as disability, ASD, attention deficit hyperactivity disorder, etc. [10,43,44,45]. The results show that study participants from the European continent obtain the highest scores when rating teacher training and parental awareness of vaccines, as most countries in Europe are among those with the highest per capita income [46]. This is closely related to greater access to vaccines, better training, and more efficient design of vaccination plans. These data are in line with current COVID-19 vaccination data worldwide, where Europe has the highest vaccination rate [46].

It is important to point out that health education requires adequate prevention to promote healthy lifestyles and limit, as far as possible, the appearance of existing diseases and comorbidities. The results obtained by age group for the assessment of teacher training and parental awareness of vaccination show that those under 30 years of age obtain the highest scores, which contrasts with the lower predisposition to be vaccinated shown by younger people in other studies [43,47]. However, this coincides with the research carried out by Kreitzman [48], on the importance of health promotion in the workplace, with those under thirty years of age being the group that most demands training in health education and vaccination of teachers working at compulsory levels.

It should be noted that, as various ethnographic studies have shown [49], health is influenced by the social, economic, and cultural context [50]. Therefore, it is necessary to intervene in the context based on cultural identities and promoting healthy lifestyle habits. Consequently, it is essential that in countries with emerging, vulnerable, and low-income economies comprehensive policies of broad scope are implemented, given that their members lack resources and education, which reduces their capacity to overcome their vulnerability and increases social and economic inequalities.

Health education in the educational system is proposed as a specific topic in which health contents and models of healthy living that imply significant changes in health-related behaviors and in the formation of values oriented to the integral development of the personality are worked on. Logically, in order to achieve these objectives, teachers must be properly trained, classroom ratios must be lowered, and schools must be provided with the necessary resources. This requires the collaboration and training of families. In this line, it is worth mentioning the approval of different programs being developed in several EU countries offering various strategies to intervene and support adherence to vaccination [51,52].

This study has shown that the participants in the group: women, under 30 years of age, HDI I1 = Very High, from the European continent and from the educational sector, are the ones who most value the fact that parents know the consequences that their children may suffer when living with other unvaccinated children. It coincides with other research [53,54,55,56] on the need to improve the education provided to parents, enhance advertising campaigns and, at the same time, train teachers and health professionals to provide information on the importance of vaccination to families. Like the research conducted by Figueroa-Almaraz et al. [57], the importance of generating greater trust between teachers, families, and health personnel by providing truthful and complete information on vaccination is valued.

## 5. Conclusions

The results of the research highlight that education cannot be configured with the same approaches and tools of the twentieth century, because the new framework generated in the fourth industrial revolution has been creating new practices, forms of interaction, communication, and a greater sense of solidarity, commitment, and responsibility of all to achieve learning objectives.

In the research carried out, all respondents considered that teachers at mandatory levels should receive initial training in health education and vaccination, with the most favorable profile being woman, under 30 years of age, educational sector, very high HDI and European continent. There was evidence of the need for teachers to be properly trained to be facilitators of contexts that promote change and, in addition, facilitate the development of reflective and critical thinking so that all people have the necessary training to control their own health and discern scientific information from possible hoaxes or interested manipulations of reality [58].

The research has shown that a large part of the population has understood the need for vaccination from the earliest age and the need for teachers to be adequately trained on the priority of vaccination as the main measure for prevention and reduction of preventable diseases. Therefore, it is evident that teachers must be adequately trained to face the new challenges posed by the methodology of health education. It involves the use of space, time, and human and material resources, as well as communication relations, so that educational strategies are implemented in the different areas of action and relationship with health services. The aim is to progressively achieve, from the first educational levels, greater autonomy, and personal empowerment, in terms of health decision-making, as well as knowledge of the personal and social determinants of health. Likewise, the importance of the involvement of the entire community for interventions to be effective [59].

In short, it is necessary to emphasize that health is one of the essential values for society, so it is necessary to empower individuals and communities so that they can increase control over the determinants of health.

This study had several limitations. The non-probabilistic consecutive sampling or total enumerative sampling used until the desired sample size was reached decreases the external validity of the research. It should be noted that the elements of the sample did not meet the conditions established to be considered a normal distribution. Indeed, the distribution by sex, age, sector, HDI, continent, and country of the sample was unequal. Consequently, the bootstrap technique was used.

Similarly, another limitation that affected data collection was the coincidence with the onset of the COVID-19 pandemic. Therefore, the evolution of the processes linked to vaccination could have influenced the transformation of the opinions and perceptions of the participants in relation to the items of the questionnaire applied.

It is also necessary to include as a limitation of the study that the HDI could be weak in profiling and distinguishing respondents, because the socioeconomic status of the participants may not have a precise correspondence with the HDI established for their country of origin.

During the research process, several proposals for improvement became evident, such as expanding some questions related to previous training in health education. Likewise, stratified probability sampling should be considered to try to homogenize the variables in the sample population.

## Figures and Tables

**Table 1:** Characteristics of the sample according to Sex, Age, HDI, Sector and Continent.

Characteristics of the Sample										
Sex	Woman		Man							
	N	%	N	%						
	694	69.4%	306	30.6%						
Age	<30		30–44		45–59		>60			
	N	%	N	%	N	%	N	%		
	363	36.30%	348	34.80%	267	26.76%	22	2.20%		
HDI	Very high		High		Middle		Low			
	N	%	N	%	N	%	N	%		
	873	87.3%	85	8.5%	31	3.1%	11	1.1%		
Sector	Health		Education		Economy					
	N	%	N	%	N	%				
	554	55.4%	329	32.9%	117	11.7%				
Continent	Africa		America		Asia		Europe		Oceania	
	N	%	N	%	N	%	N	%	N	%
	35	3.5%	93	9.3%	40	4%	830	83%	2	0.2%

**Table 2:** Count after the application of the questionnaire.

Dimension (D)	Item	Scale (*n*)			*n*	M	95% CI		SD	95% CI	
		1	2	3			Lower	Upper		Lower	Upper
D1 = Awareness and Regulation	P01	26	174	800	1000	2.77	2.75	2.80	0.48	0.44	0.51
	P02	14	132	854	1000	2.84	2.81	2.86	0.40	0.37	0.44
	D1t					2.81	2.78	2.83	0.36	0.34	0.38
D2 = Education and Teachers	P03	34	131	835	1000	2.80	2.77	2.83	0.48	0.44	0.52
	P04	27	152	821	1000	2.79	2.77	2.82	0.47	0.43	0.50
	P05	35	139	826	1000	2.79	2.76	2.82	0.49	0.44	0.52
	P06	35	74	891	1000	2.86	2.83	2.88	0.44	0.40	0.49
	D2t					2.81	2.79	2.83	0.31	0.28	0.33
D3 = Regulation and Obligation	P07	616	183	201	1000	1.59	1.53	1.64	0.80	0.78	0.83
	P08	701	240	59	1000	1.36	1.32	1.40	0.59	0.56	0.62
	D3t					1.47	1.44	1.51	0.59	0.56	0.61
D4 = Consequences and Risks	P09	791	174	35	1000	1.24	1.21	1.28	0.50	0.47	0.54
	P10	828	149	23	1000	1.20	1.17	1.22	0.45	0.41	0.48
	P11	840	143	17	1000	1.18	1.15	1.20	0.42	0.39	0.46
	P12	836	147	17	1000	1.18	1.16	1.21	0.43	0.39	0.46
	D4t					1.20	1.18	1.23	0.41	0.37	0.44
Total	S3t					2.05	2.04	2.06	0.22	0.20	0.23

**Table 3:** Count by Sex of the participating sample for dimension D2.

Dimension (D)	Item	Sex						*n*
		**Man** ***n* = 306 (30.60%)**			**Woman** ***n* = 694 (69.4%)**			
		1	2	3	1	2	3	
D2 = Education and Teachers	P03	15	50	241	19	81	594	1000
	P04	12	54	240	15	98	581	1000
	P05	14	52	240	21	87	586	1000
	P06	13	31	262	22	43	629	1000

**Table 4:** ANOVA for Sex-independent samples for dimension D2.

Dimension (D)	Item	M	Man	SD	95% CI	M	Woman	SD	95% CI	F	*p*	Eta2	Direction
			L-U		L-U		L-U		L-U				
D2 Education and Teachers	P03	2.74	2.68–2.79	0.54	0.47–0.60	2.83	2.80–2.86	0.44	0.39–0.49	7.60	0.01	0.01	W > M
	P04	2.75	2.68–2.80	0.52	0.45–0.58	2.82	2.78–2.85	0.44	0.40–0.48	4.86	0.03	0.00	W > M
	P05	2.74	2.67–2.80	0.53	0.46–0.60	2.81	2.78–2.85	0.46	0.41–0.51	5.17	0.02	0.01	W > M
	P06	2.81	2.76–2.87	0.49	0.41–0.56	2.87	2.84–2.90	0.42	0.36–0.47	4.09	0.04	0.00	W > M
	D2t	2.76	2.72–2.79	0.33	0.29–0.36	2.83	2.81–2.86	0.29	0.26–0.32	12.57	0.00	0.01	W > M

Note: L = Lower; U = Upper.

**Table 5:** Count by age group of the participating sample for dimension D2.

Dimension (D)	Item	Age												*n*
		<30 *n* = 363 (36.3%)			Between 31–44 *n* = 348 (34.8%)			Between 45–59 *n* = 267 (26.76%)			>60 *n* = 22 (2.2%)			
		1	2	3	1	2	3	1	2	3	1	2	3	
D2 = Education and Teachers	P03	10	21	332	10	63	275	11	44	212	3	3	16	1000
	P04	6	22	335	9	70	269	10	52	205	2	8	12	1000
	P05	10	25	328	9	59	280	12	52	203	4	3	15	1000
	P06	7	11	345	16	34	298	11	28	228	1	1	20	1000

**Table 6:** ANOVA for independent samples by age group for dimension D2.

**Item**	**M**	**E1 ≤ 30**	**SD**	**95% CI**	**M**	**E2 = 30–44**	**SD**	**95% CI**	***p***	**Eta2**	**Direction**
P03	2.89	2.84–2.93	0.39	0.31–0.47	2.76	2.71–2.81	0.49	0.43–0.55	<0.01	0.02	E1 > E2.E3
P04	2.91	2.87–2.94	0.34	0.26–0.41	2.75	2.69–2.80	0.49	0.44–0.54	<0.01	0.04	E1 > E2.E3.E4
P05	2.88	2.83–2.92	0.41	0.33–0.48	2.78	2.73–2.83	0.47	0.41–0.53	<0.01	0.03	E1 > E2.E3
P06	2.93	2.90–2.96	0.32	0.22–0.40	2.81	2.76–2.86	0.50	0.42–0.56	<0.01	0.02	E1 > E2.E3
D2t	2.90	2.87–2.92	0.25	0.20–0.29	2.77	2.74–2.81	0.33	0.30–0.37	<0.01	0.06	E1 > E2.E3.E4
**Item**	**M**	**E3 = 45–59**	**SD**	**95% CI**	**M**	**E4 ≥ 60**	**SD**	**95% CI**	***p***	**Eta2**	**Direction**
P03	2.75	2.69–2.81	0.52	0.44–0.59	2.59	2.26–2.87	0.73	0.36–0.92	<0.01	0.02	E1 > E2.E3
P04	2.73	2.66–2.79	0.52	0.46–0.59	2.45	2.16–2.73	0.67	0.47–0.83	<0.01	0.04	E1 > E2.E3.E4
P05	2.72	2.65–2.78	0.54	0.47–0.61	2.50	2.16–2.82	0.80	0.50–0.96	<0.01	0.03	E1 > E2.E3
P06	2.81	2.75–2.87	0.49	0.39–0.56	2.86	2.63–3.00	0.47	0.00–0.75	<0.01	0.02	E1 > E2.E3
D2t	2.75	2.72–2.79	0.30	0.27–0.33	2.60	2.41–2.78	0.43	0.26–0.55	<0.01	0.06	E1 > E2.E3.E4

Note: L = Lower; U = Upper.

**Table 7:** Count by sector of the participating sample for dimension D2.

Dimension (D)	Item	Sector									*n*
		Health *n* = 554 (55.4%)			Education *n* = 329 (32.9%)			Economy *n* = 117 (11.7%)			
		1	2	3	1	2	3	1	2	3	
D2 = Education and teachers	P03	33	96	425	0	22	307	1	13	103	1000
	P04	23	105	426	0	29	300	4	18	95	1000
	P05	30	95	429	1	22	306	4	22	91	1000
	P06	24	55	475	5	7	317	6	12	99	1000

**Table 8:** ANOVA for sector-independent samples for dimension D2.

**Item**	**M**	**S1 = Health**	**SD**	**95% CI**	***p***	**Eta2**	**Direction**
P03	2.71	2.66–2.76	0.57	0.52–0.62	<0.01	0.05	S2.S3 > S1
P04	2.73	2.68–2.77	0.53	0.48–0.58	<0.01	0.03	S2 > S1.S3
P05	2.72	2.67–2.77	0.56	0.50–0.61	<0.01	0.04	S2 > S1.S3
P06	2.81	2.77–2.86	0.49	0.43–0.54	<0.01	0.02	S2 > S1.S3
D2t	2.74	2.71–2.77	0.35	0.32–0.37	<0.01	0.08	S2 > S1.S3
**Item**	**M**	**S2 = Education**	**SD**	**95% CI**	***p***	**Eta2**	**Direction**
P03	2.93	2.91–2.96	0.25	0.20–0.29	<0.01	0.05	S2.S3 > S1
P04	2.91	2.88–2.94	0.28	0.24–0.33	<0.01	0.03	S2 > S1.S3
P05	2.93	2.89–2.95	0.27	0.21–0.33	<0.01	0.04	S2 > S1.S3
P06	2.95	2.92–2.98	0.28	0.17–0.37	<0.01	0.02	S2 > S1.S3
D2t	2.93	2.91–2.95	0.17	0.14–0.20	<0.01	0.08	S2 > S1.S3
**Item**	**M**	**S3 = Economy**	**SD**	**95% CI**	***p***	**Eta2**	**Direction**
P03	2.87	2.80–2.93	0.36	0.26–0.45	<0.01	0.05	S2.S3 > S1
P04	2.78	2.68–2.86	0.49	0.38–0.60	<0.01	0.03	S2 > S1.S3
P05	2.74	2.64–2.83	0.51	0.40–0.61	<0.01	0.04	S2 > S1.S3
P06	2.79	2.69–2.89	0.52	0.37–0.62	<0.01	0.02	S2 > S1.S3
D2t	2.80	2.74–2.85	0.30	0.23–0.36	<0.01	0.08	S2 > S1.S3

**Table 9:** Count by Human Development Index (HDI) for dimension D2.

Dimension (D)	Item	HDI												*n*
		Very High *n* = 873 (87.3%)			High *n* = 85 (8.5%)			Medium *n* = 31 (3.1%)			Low *n* = 11 (1.1%)			
		1	2	3	1	2	3	1	2	3	1	2	3	
D2 = Education and teachers	P03	14	90	769	10	29	46	8	4	19	2	8	1	1000
	P04	16	104	753	8	28	49	3	10	18	0	10	1	1000
	P05	22	109	742	8	19	58	4	5	22	1	6	4	1000
	P06	24	56	793	7	12	66	3	3	25	1	3	7	1000

**Table 10:** ANOVA for independent samples by Human Development Index for dimension D2.

**Item**	**M**	**I1 = Very High**	**SD**	**95% CI**	**M**	**I2 = High**	**SD**	**95% CI**	***p***	**Eta2**	**Direction**
P03	2.86	2.84–2.89	0.39	0.34–0.43	2.42	2.28–2.57	0.70	0.60–0.78	<0.01	0.13	I1 > I2 > I3 > I4
P04	2.84	2.82–2.87	0.41	0.37–0.45	2.48	2.34–2.62	0.67	0.56–0.75	<0.01	0.09	I1 > I2.I3 > I5
P05	2.82	2.80–2.85	0.44	0.40–0.48	2.59	2.45–2.71	0.66	0.53–0.76	<0.01	0.04	I1 > I2.I3 > I4
P06	2.88	2.85–2.91	0.40	0.35–0.45	2.69	2.56–2.82	0.62	0.46–0.73	<0.01	0.02	I1 > I3 > I2 > I4
D2t	2.85	2.84–2.87	0.26	0.24–0.29	2.55	2.47–2.63	0.41	0.35–0.46	<0.01	0.15	I1 > I2 > I3 > I4
**Item**	**M**	**I3 = Medium**	**SD**	**95% CI**	**M**	**I4 = Low**	**SD**	**95% CI**	***p***	**Eta2**	**Direction**
P03	2.35	2.04–2.68	0.88	0.66–0.97	1.91	1.58–2.22	0.54	0.00–0.76	<0.01	0.13	I1 > I2 > I3 > I4
P04	2.48	2.23–2.71	0.68	0.49–0.82	2.09	2.00–2.31	0.30	0.00–0.49	<0.01	0.09	I1 > I2.I3 > I5
P05	2.58	2.32–2.81	0.72	0.48–0.87	2.27	1.88–2.67	0.65	0.38–0.85	<0.01	0.04	I1 > I2.I3 > I4
P06	2.71	2.46–2.91	0.64	0.31–0.83	2.55	2.08–2.89	0.69	0.32–0.93	<0.01	0.02	I1 > I3 > I2 > I4
D2t	2.53	2.40–2.67	0.37	0.30–0.44	2.20	1.98–2.42	0.37	0.19–0.48	<0.01	0.15	I1 > I2 > I3 > I4

**Table 11:** Count by Continent of the participating sample for dimension D2.

Dimension (D)	Item	Continent															*n*
		Europe *n* = 830 (83%)			America *n* = 93 (9.3%)			Asia *n* = 40 (4%)			Africa *n* = 35 (3.5%)			Oceania *n* = 2 (2%)			
		1	2	3	1	2	3	1	2	3	1	2	3	1	2	3	
D2 = Education and Teachers	P03	13	84	733	4	29	60	10	5	25	7	12	16	0	1	1	1000
	P04	15	99	716	3	28	62	7	5	28	2	20	13	0	0	2	1000
	P05	20	102	708	4	23	66	9	3	28	2	11	22	0	0	2	1000
	P06	24	50	756	4	16	73	3	3	34	4	4	27	0	1	1	1000

**Table 12:** ANOVA for independent samples by Continent for dimension D2.

**Item**	**M**	**C1 = Europe**	**SD**	**95% CI**	**M**	**C2 = America**	**SD**	**95% CI**	***p***	**Eta2**	**Direction**
P03	2.87	2.84–2.89	0.38	0.34–0.42	2.60	2.47–2.71	0.57	0.48–0.66	<0.01	0.11	C1 > C2.C3.C4
P04	2.84	2.82–2.87	0.41	0.37–0.45	2.63	2.53–2.74	0.55	0.46–0.63	<0.01	0.07	C1 > C2.C4
P05	2.83	2.80–2.86	0.44	0.39–0.47	2.67	2.54–2.77	0.56	0.45–0.65	<0.01	0.04	C1.C2.C3.C4
P06	2.88	2.85–2.91	0.40	0.35–0.45	2.74	2.63–2.84	0.53	0.40–0.64	<0.01	0.02	C1.C2.C3.C4
D2t	2.86	2.84–2.87	0.26	0.24–0.28	2.66	2.58–2.73	0.38	0.32–0.44	<0.01	0.12	C1 > C2.C3.C4
**Item**	**M**	**C3 = Asia**	**SD**	**95% CI**	**M**	**C4 = Africa**	**SD**	**95% CI**	***p***	**Eta2**	**Direction**
P03	2.38	2.10–2.63	0.87	0.71–0.96	2.26	1.97–2.52	0.78	0.63–0.88	<0.01	0.11	C1 > C2.C3.C4
P04	2.53	2.28–2.76	0.78	0.55–0.91	2.31	2.12–2.50	0.58	0.47–0.68	<0.01	0.07	C1 > C2.C4
P05	2.48	2.21–2.73	0.85	0.64–0.95	2.57	2.35–2.77	0.61	0.44–0.75	<0.01	0.04	C1.C2.C3.C4
P06	2.78	2.60–2.94	0.58	0.29–0.76	2.66	2.44–2.88	0.68	0.42–0.84	<0.01	0.02	C1.C2.C3.C4
D2t	2.54	2.42–2.65	0.39	0.32–0.47	2.45	2.30–2.60	0.45	0.36–0.52	<0.01	0.12	C1 > C2.C3.C4

**Table 13:** Spearman matrix of nonparametric bivariate correlations.

Variables	Significance	AGE	SEX	HDI	CONT.	SECTOR
AGE	Correlation Coefficient	1.000	0.234 **	0.206 **	0.256 **	−0.209 **
	Sig. (2-tailed)		<0.001	<0.001	<0.001	<0.001
SEX	Correlation Coefficient	0.234 **	1.000	0.120 **	0.127 **	−0.096 **
	Sig. (2-tailed)	<0.001		<0.001	<0.001	0.002
HDI	Correlation Coefficient	0.206 **	0.120 **	1.000	0.813 **	−0.193 **
	Sig. (2-tailed)	<0.001	<0.001		<0.001	<0.001
SECTOR	Correlation Coefficient	−0.209 **	−0.096 **	−0.193 **	−0.211 **	1.000
	Sig. (2-tailed)	<0.001	0.002	<0.001	<0.001	
CONT.	Correlation Coefficient	0.256 **	0.127 **	0.813 **	1.000	−0.211 **
	Sig. (2-tailed)	<0.001	<0.001	<0.001		<0.001

** Correlation is significant at the 0.01 level (2-tailed).

## Data Availability

Due to the anonymity and confidentiality of the data obtained, the authors have not reported any of the data obtained, the purpose of which is exclusively the development of this research.
